# *Disc1* Carrier Mice Exhibit Alterations in Neural pIGF-1Rβ and Related Kinase Expression

**DOI:** 10.3389/fncel.2020.00094

**Published:** 2020-05-05

**Authors:** Razia Sultana, Amita Shrestha, Charles C. Lee, Olalekan M. Ogundele

**Affiliations:** Department of Comparative Biomedical Sciences, Louisiana State University School of Veterinary Medicine, Baton Rouge, LA, United States

**Keywords:** *disc1*, disease models, cognition, pIGF-1Rβ, Erk1/2, GSK3β

## Abstract

Mutation of the *disc1* gene underlies a broad range of developmental neuropsychiatric defects, including schizophrenia, depression, and bipolar disorder. The pathophysiological phenotypes linked with *disc1* mutation are due to the truncation of the DISC1 primary protein structure. This leads to a defective post-synaptic scaffolding and kinase—GSK3β and Erk1/2—signaling. As a result, synaptic function and maintenance are significantly impaired in the *disc1* mutant brain. Among several other pathways, GSK3β and Erk1/2 are involved in insulin-like growth factor 1 receptor (IGF-1Rβ) kinase signaling. Although *disc1* mutation alters these kinases, it is unclear if the mutation impacts IGF-1R expression and activity in the brain. Here, we demonstrate that the expression of active IGF-1Rβ (pIGF-1Rβ) is altered in the hippocampus and prefrontal cortex (PFC) of *disc1* mutant mice and vary with the dose of the mutation (homozygous and heterozygous). The expression of pIGF-1Rβ decreased significantly in 129S (*hom, disc1*^−/−^) brains. In contrast, 129S:B6 (*het, disc1*^+/−^) brains were characterized by an increase in pIGF-1Rβ when compared with the C57BL/6 (*disc1*^+/+^) level. The decrease in pIGF-1Rβ level for the 129S brains was accompanied by the loss of Akt activity (S473 pAkt) and decreased Ser9 phosphorylation of GSK3β (increased basal GSK3β). Additionally, hippocampal and cortical pErk1/2 activity increased in the 129S hippocampus and cortex. Although 129S:B6 recorded alterations in pIGF-1Rβ-pAkt-GSK3β (like 129S), there was no observable change in pErk1/2 activity for the heterozygote (*disc1*^+/−^) mutant. In addition to GSK3β inhibition, we conclude that pIGF-1R, pAkt, and pErk1/2 are potential targets in *disc1*^−/−^ mutant brain. On the other hand, pIGF-1R and pAkt can be further explored in *disc1*^+/−^ brain.

## Introduction

In humans, *disc1* gene mutation is an associative cause of a broad range of developmental neuropsychiatric disorders (Clapcote and Roder, 2006; Koike et al., 2006; Ross et al., 2006; Kvajo et al., 2008; Brandon et al., 2009; Soares et al., 2011; Wang et al., 2011; Wexler and Geschwind, 2011; Zheng et al., 2011; Gómez-Sintes et al., 2014). Neuropsychiatric conditions resulting from *disc1* mutation are attributable to the involvement of the gene product, DISC1 protein, in neurodevelopment, synaptogenesis, neurite outgrowth, neurotransmitter signaling, and synaptic plasticity (Koike et al., 2006; Ross et al., 2006; Brandon et al., 2009; Kim et al., 2009; Lee et al., 2011a; Ramsey et al., 2011; Wexler and Geschwind, 2011; Dachtler et al., 2016; Tomoda et al., 2016). DISC1 is a regulatory post-synaptic scaffolding protein that is linked to kinase signaling, cytoskeleton, and excitatory neurotransmitter receptors (Ross et al., 2006; Kvajo et al., 2008; Ramsey et al., 2011). Notably, DISC1 is involved in the scaffolding of post-synaptic N-Methyl-D-Aspartate Receptor 1 through its interaction with the GluN1 and GluN2B subunits. As a result of this interaction, DISC1 directs the translocation of NMDAR to the post-synaptic membrane and controls aspects of plasticity (Malavasi et al., 2018). Mutation of the *disc1* gene leads to a truncation of the DISC1 primary protein structure and is accompanied by an incremental loss of NMDAR function (Ramsey et al., 2011; Wexler and Geschwind, 2011; Snyder and Gao, 2013; Malavasi et al., 2018). This underlies long-term potentiation (LTP) defects that lead to spine dysgenesis and cognitive decline. As such, the neural changes caused by *disc1* mutations produce behavioral phenotypes that are characteristic of neuropsychiatric disorders with associative NMDAR hypofunction (Koike et al., 2006; Kvajo et al., 2008; Lee et al., 2011a,b; Lipina et al., 2011; Namba et al., 2011; Ramsey et al., 2011; Snyder and Gao, 2013; Gómez-Sintes et al., 2014; Tomoda et al., 2016; Shao et al., 2017; Malavasi et al., 2018).

DISC1 protein signaling regulates the synaptic activity of GSK3β (Kvajo et al., 2008; Kim et al., 2009; Lee et al., 2011b; Lipina et al., 2011) and Erk1/2 (Soares et al., 2011). Owing to the role of GSK3β (Clayton et al., 2010; Hur and Zhou, 2010; Lee et al., 2011b; Emamian, 2012; Kitagishi et al., 2012; Dachtler et al., 2016; Swiatkowski et al., 2017; Wang et al., 2017) and Erk1/2 (Xia et al., 1996; Roux and Blenis, 2004; Roskoski, 2012; Xing et al., 2016; Nikolaienko et al., 2017; Ohta et al., 2017; Gao and Zhao, 2018; Iyaswamy et al., 2018; Pucilowska et al., 2018) in the control of neurodevelopment, synaptogenesis, and spine plasticity, *disc1* mutations lead to detrimental changes in synaptic function and behavior. With that said, drugs that modulate GSK3β (Lee et al., 2011b; Emamian, 2012; Bhat et al., 2018) and Erk1/2 (Lu and Dwyer, 2005; Pereira et al., 2014; Tassin et al., 2015; Aringhieri et al., 2017; Hirayama-Kurogi et al., 2017) have shown significant promise in treating synaptic and behavioral defects of schizophrenia, depression, and bipolar disorder.

In the developing nervous system, deficiency in neurotrophic factors (e.g., IGF-1, BDNF, and NGF), and a change in the expression of their associated receptors leads to dendritic spine malformations (Ohta et al., 2017; Reim and Schmeisser, 2017). Specifically, attenuation of insulin-like growth factor 1 receptor (IGF-1Rβ) kinase activity in the developing brain abrogates synaptogenesis and leads to dendritic spine loss (Lee C. C. et al., 2011; Lee et al., 2011b; González Burgos et al., 2012; Nakahata and Yasuda, 2018). This is attributable to the dysregulation of downstream kinases—GSK3β, Erk1/2, Akt/PKB—involved in the control of neuronal migration, differentiation, dendritogenesis, and structural organization within the nervous system (Nieto Guil et al., 2017; Reim and Schmeisser, 2017). Accordingly, genetic knockdown or overexpression of these kinases leads to abnormalities in dendrite morphology, synaptic pruning, and behavior (Wan et al., 2007; Del’Guidice and Beaulieu, 2010; Lee C. C. et al., 2011; Emamian, 2012; Kitagishi et al., 2012; Wang et al., 2017).

Although *disc*1 *mutation* promulgates erroneous GSK3β and Erk1/2 activity, the impact on pIGF-1Rβ expression and activity is yet to be investigated in the cognitive centers. Erk1/2 and GSK3β are downstream effector molecules of pIGF-1Rβ kinase activity and are involved in the maintenance of the synaptic structure. GSK3β and Erk1/2 activity are also pertinent to the propagation of LTP, and coupling of synaptic function to cellular regulation (Peineau et al., 2007; Dewachter et al., 2009; Vara et al., 2009; Giachello et al., 2010; Shahab et al., 2014). Downstream of pIGF-1Rβ, Erk1/2 (Roux and Blenis, 2004; Roskoski, 2012) and GSK3β (Hur and Zhou, 2010) are involved related pathways that regulates cell proliferation and cell survival. As such, alteration in the activity of these kinases in *disc1* mutation may disrupt signaling cascades that involve pIGF-1Rβ.

The study provides evidence of pIGF-1Rβ dysregulation in the hippocampus and prefrontal cortex (PFC) of mutant *disc1* carrier mice. In addition to changes in neural GSK3β and Erk1/2 expression, heterozygous *129S:B6* (*disc1*^+/−^) and homozygous *129S* (*disc1*^−/−^) carriers exhibit a change in neural pIGF-1Rβ expression. Here, we show some of the differences and similarities in the pattern of pIGF-1Rβ dysregulation for the hippocampus and PFC of these* disc1* carrier mice.

## Materials and Methods

The 129S (*disc1^−/−^)* mice (RRID:IMSR_JAX:002448) were acquired from the Jackson Lab (Bar Harbor, ME, United States) and have a spontaneous C-terminal truncation mutation in the* disc1* gene (Clapcote and Roder, 2006). The 129S:B6 (*disc1*^+/−^) line (RRID:IMSR_JAX:101043) is from a cross of the 129S and C57BL/6J mouse lines. For comparison, we used the C57BL/6J (B6) line (RRID:IMSR_JAX:000664) as carriers of the wild-type* disc1* gene (*disc1*^+/+^). We have previously demonstrated that 129S mice vary behaviorally from all other inbred strains, including the C57BL/6J, and have phenotypes that are similar to other *disc1* knockout strains (Sultana et al., 2019). Animals were housed under standard laboratory conditions of 12 h alternating light and dark cycle with food and water provided *ad libitum*. All animal handling procedures were approved by the Institutional Animal Care and Use Committee (IACUC) of the Louisiana State University School of Veterinary Medicine. Adult mice (PND 90–100) weighing between 22–26 g were used for this study (C57BL/6J: *n* = 9, 129S:B6: *n* = 9; 129S: *n* = 10).

### Specimen Preparation

Mice were euthanized in an isoflurane chamber. Subsequently, the animals were transcardially perfused with 10 mM PBS (pH 7.4). The whole brain was harvested and rapidly placed in cold artificial cerebrospinal fluid (aCSF) maintained on ice, and saturated with 95% Oxygen/5%CO_2_. A clean razor blade was used to cut the brain—along the sagittal plane—into two (left and right) hemispheres. The left and right hemispheres were microdissected, and the hippocampus was extracted by exposing the space between the cortex and corpus callosum. A surgical blade was used to cut the PFC. The harvested hippocampal and prefrontal cortical tissue was kept in separate tubes and stored at −80°C until further use.

### Immunoblotting

Frozen hippocampal and prefrontal cortical tissue were incubated on ice with RIPA lysis cocktail containing protease and phosphatase inhibitors. After 30 min, the incubated tissue was rapidly homogenized to obtain tissue lysate. This was further centrifuged to obtain a supernatant containing cytoplasmic, membrane and synaptic fragments (whole lysate). To enrich synaptosomes, we used a previously established Sucrose-HEPES gradient technique (Kamat et al., 2014; Tenreiro et al., 2017). Ten microliter whole lysate or 4 μl synaptosomal extract containing 10 μg of protein was processed for SDS-PAGE electrophoresis (C57BL/6J: *n* = 4, 129S:B6: *n* = 4; 129S: *n* = 5). After western blotting (wet transfer), Polyvinylidene fluoride membrane (PVDF) was incubated in Tris-buffered saline (with 0.01% Tween 20) for 15 min (i.e., TBST) at room temperature. Afterward, the membrane was blocked in 3% bovine serum albumin (prepared in TBST) for 50 min at room temperature. The protein of interest and house-keeping protein were detected using the following primary antibodies; Rabbit anti-GSK-3β (Cell Signaling #12456S), Rabbit anti-Phospho-GSK-3α/β:Ser21/9 (Cell Signaling #9331S), Rabbit anti-Phospho-Erk1/Erk2:Thr185/Tyr187 (Thermofisher Scientific ABfinity™ Antibody #700012), Rabbit anti Erk1/2 Antibody (Thermofisher Scientific # PA1-4703), Rabbit anti-Phospho-IGF1Rβ:Tyr1161 Antibody (Thermofisher Scientific #PA5-37601), Rabbit anti-Phospho-IGF1R/Insulin Receptor β:Tyr1131/1146 Antibody (Cell Signaling #3021S), Rabbit anti-IGF1-Rβ (Cell Signaling #3027S), Rabbit anti-Akt (Cell Signaling #9272S), and Rabbit anti-Phospho Akt: Ser473 (Cell Signaling #4060S). All primary antibodies were diluted in the blocking solution at 1:1,000. Subsequently, the primary antibodies were detected using Chicken anti-Rabbit-HRP secondary antibody (Thermofisher Scientific #A15987) at a dilution of 1:5,000 or 1:10,000. The reaction was developed using a chemiluminescence substrate (Thermofisher-#34579). To normalize protein expression, the membranes were treated with Restore PLUS Western Blot Stripping Buffer (Thermofisher Scientific #46430) and re-probed with β-Actin (8H10D10) Mouse mAb HRP Conjugate (Cell Signaling #12262S). Protein expression was normalized *per lane* using the corresponding β-Actin expression. At least two repeats were performed for each of the proteins quantified by immunoblotting. Protein expression for the experimental groups was compared using One-Way ANOVA with Tukey *post hoc* test. Significance was also confirmed using the Kruskal-Wallis test (GraphPad Prism version 8.0). Here, we have presented One-Way ANOVA outcomes as bar charts with error bars depicting the mean and standard error of mean, respectively.

### Immunofluorescence

After perfusion with 10 mM PBS (pH 7.4), the whole-brain was fixed in 4% phosphate-buffered paraformaldehyde (PB-PFA) overnight, and then transferred to 4% PB-PFA containing 30% sucrose for cryopreservation. Free-floating cryostat sections (20 μm) were obtained and preserved in 48-well plates containing 10 mM PBS at 4°C (C57BL/6J: *n* = 5, 129S:B6: *n* = 5; 129S: *n* = 5). The sections were washed three times (5 min each) in 10 mM PBS (pH 7.4) on a slow orbital shaker. Subsequently, non-specific blocking was performed in either 5% normal goat serum (Vector Labs #S-1000), chicken serum (Abcam #ab7477) or donkey Serum (Abcam #ab7475), prepared in 10 mM PBS + 0.03% Triton-X100, for 1 h at room temperature. The sections were incubated overnight at 4°C in primary antibody diluted in blocking solution (10 mMPBS+0.03% Triton-X 100 and 5% normal goat, chicken or donkey serum). The following primary antibodies were used for this procedure; Rabbit anti-Phospho-GSK-3α/β:Ser21/Ser9 (Cell Signaling #9331S), Rabbit anti-Phospho-IGF-1R beta:Tyr1161 Antibody (Thermofisher Scientific #PA5-37601), and Rabbit anti-NeuN Alexa-488 Conjugate (EMD Millipore #MAB377XMI MI). Subsequently, the sections were washed two times in 10 mM PBS in preparation for secondary antibody incubation. The sections were subsequently incubated in Goat anti-Rabbit Alexa 568 (Thermofisher Scientific #A-11036) secondary antibody for 1 h at room temperature, with gentle shaking (35rpm). Immunolabeled sections were washed and mounted on gelatin-coated slides using ProLong™ Diamond Antifade Mountant containing DAPI (Thermofisher Scientific #P36971).

### Quantification

Fluorescence imaging was performed using a Nikon-*NiU* fluorescence upright microscope configured for 3D imaging. Z-stacks were obtained and converted into 2D images through the extended depth focus (EDF) option on Nikon Element software. Normalized fluorescence intensity for immunolabeled proteins in the hippocampus and medial PFC was performed in optical slices for serial section images (*n = 5* per group). Fluorescence intensity was quantified using Nikon Element AR. Mean cell count and intensity were determined per unit area in several fields of view for consecutive sections. Fluorescence intensity was normalized by applying a uniform exposure time for a fluorophore-labeled protein in the control and test brain slices.

### Statistical Analysis

Statistical comparison between C57BL/6J (*disc1^+/+^*), 129S:B6 (*disc1*^+/−^), and *129S* (*disc1*^−/−^), protein expression and fluorescence intensity were determined using one-way ANOVA with Tukey *post hoc* test. Significance was also confirmed using the Kruskal-Wallis test (GraphPad Prism version 8.0). Here, we have presented one-way ANOVA outcomes as bar charts with error bars depicting the mean and standard error of mean, respectively.

## Results

**Figure 1:**
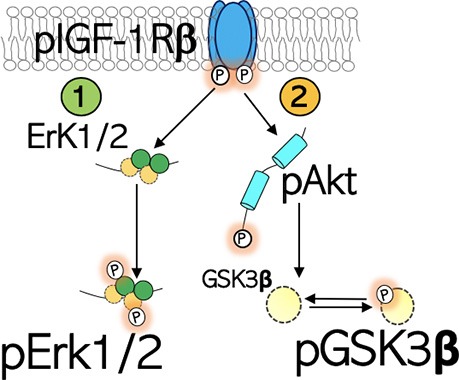
Schematic illustration of the IGF-1R signaling. IGF-1Rβ is activated by phosphorylation of the Tyrosine (Tyr) residue of the kinase domain. (1) pIGF-1Rβ kinase signaling increases Erk1/2 phosphorylation and activity. This signaling pathway directs cell proliferation. (2) pIGF-1Rβ kinase signaling facilitates Ser473 phosphorylation of Akt (pAkt), which in turn phosphorylates (inactivates) GSK3β at Ser9 site (pGSK3β).

Differential dysregulation of neural pIGF-1Rβ activity occurred in the hippocampus and PFC of the 129S:B6 (*disc1^+/−^*) and 129S (*disc*^−/−^) mice. Truncation of the primary structure of synaptic scaffolding protein, DISC1, leads to an increase in GSK3β signaling (Lee et al., 2011b; Lipina et al., 2011) and altered Erk1/2 signaling (Soares et al., 2011). Mechanistically, pIGF-1Rβ kinase signaling increases Akt-PKB phosphorylation (pAkt), which in turn phosphorylates (inactivates) GSK3β (Wan et al., 2007; Del’Guidice and Beaulieu, 2010; Chandarlapaty et al., 2011; Wang et al., 2017). Thus, in the 129S:B6 and 129S brain, we determined whether a change in GSK3β and ErK1/2 activity that is linked to disc1 mutation also involves alterations in neural pIGF-1Rβ level (Figure 1). While the *disc1* gene mutation caused a change in neural pIGF-1Rβ activity, the pattern of dysregulation varied with the dose of the mutation. The 129S:B6 hippocampus and PFC recorded an increase in pIGF-1Rβ expression when compared with the C57BL/6J. In contrast, the 129S hippocampus and PFC exhibited a loss of neural pIGF-1Rβ vs. the C57BL/6J and 129S:B6.

Subsequent analysis of Akt, GSK3β, and Erk1/2 expression showed that a decreased pIGF-1Rβ level in the 129S brain may be related to DISC1 loss of function. As such, in the *disc1*^−/−^ brain, a decrease in pIGF-1Rβ was accompanied by a loss of pAkt (Ser473), increased basal GSK3β activity, and a general increase in pErk1/pErk2 activity.

### pIGF-1Rβ

**Figure 2:**
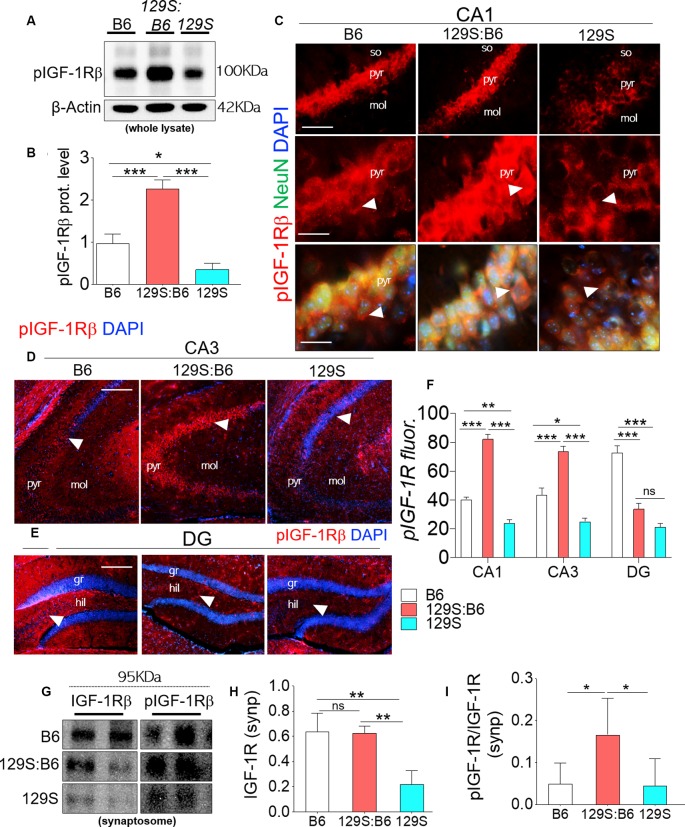
Expression of pIGF-1Rβ in B6 (*disc1*^+/+^), 129S:B6 (*disc1*^+/−^), and 129S (*disc1*^−/−^), hippocampus. **(A)** Representative western blots demonstrating pIGF-1Rβ expression in B6, 129S:B6 and 129S hippocampal whole lysate. **(B)** One-way ANOVA comparison of β-actin normalized pIGF-1Rβ expression. **(C)** Representative immunofluorescence images showing the expression of pIGF-1Rβ in the CA1 (scale bar = 60 μm and 40 μm). **(D,E)** Representative immunofluorescence images showing the expression of pIGF-1Rβ in the CA3, and DG regions of the hippocampus (scale bar = 80 μm; so, stratum oriens; pyr, pyramidal layer; mol, molecular layer; gr, granular layer; and hil: hilus of dentate gyrus). **(F)** Bar graph illustrating statistical comparison for pIGF-1Rβ fluorescence intensity in the CA1, CA3, and DG. **(G)** Representative western blots demonstrating IGF-1Rβ and pIGF-1Rβ expression in B6, 129S:B6 and 129S hippocampal synaptosomal (synp) extracts. **(H,I)** A statistical representation of normalized synaptosomal IGF-1Rβ and pIGF-1Rβ expression [(*n* = 4 to *n* = 6 per group; **B,F,H,I**) *p < 0.05, **p < 0.01, ***p < 0.001; ns, no significance].

pIGF-1Rβ expression was significantly downregulated in 129S hippocampal whole lysates when compared with B6 (*p* < 0.05) and 129S:B6 (*p* < 0.001; Figures 2A,B). Interestingly, 129S:B6 hippocampus recorded an increase in pIGF-1Rβ expression when compared with the control (*p* < 0.001). To verify this outcome, we assessed pIGF-1Rβ expression in CA1, CA3 and DG neurons (NeuN) using immunofluorescence quantification method (Figures 2C–E). In the 129S:B6 hippocampus, increased pIGF-1Rβ protein level (Figures 2A,B) was associated with an increase in pIGF-1Rβ fluorescence for NeuN^+^ cells in the CA1 and CA3 pyramidal layers (Figure 2F; *p* < 0.001). Conversely, decreased pIGF-1Rβ expression in 129S hippocampal lysate (Figures 2A,B) was accompanied by a lower pIGF-1Rβ fluorescence in the NeuN^+^ cells of CA1 (*p* < 0.01), CA3 (*p* < 0.05), and DG (*p* < 0.001) when compared with B6 (Figure 2F, see also Supplementary Figure S1).

In a subsequent experiment, we performed immunoblotting to detect pIGF-1Rβ and IGF-1Rβ in hippocampal synaptosomal tissue extracts (Figure 2G). Our results revealed that basal IGF-1Rβ expression in hippocampal synaptosomal extracts was unaffected by the heterozygote dose of disc1 mutation (*disc1*^+/−^). When synaptosomal IGF-1Rβ expression was normalized by β-actin, there was no significant difference in comparison with the B6 mice (*disc1*^+/+^). In contrast, homozygote (*disc1*^−/−^) dose of the mutation caused a significant decrease in basal synaptosomal IGF-1Rβ level versus the B6 (*disc1*^+/+^, *p* < 0.01) and 129S:B6 (*disc1*^+/−^, *p* < 0.01; Figure 2G). In subsequent analysis, we determined IGF-1R kinase activity by normalizing pIGF-1Rβ with basal IGF-1Rβ expression (Figure 2I). Although IGF-1Rβ expression was relatively unchanged in 129S:B6 (*disc1*^+/−^) synaptosomal extracts, there was a significant increase in normalized pIGF-1Rβ which indicates an increased activity vs. the B6 (*p* < 0.05; Figure 2I). On the other hand, the observed decline in IGF-1Rβ level in the 129S hippocampal synaptosomal extracts did not translate into a change in pIGF-1Rβ activity. Thus, IGF-1Rβ normalized pIGF-1Rβ activity was unchanged when the 129S hippocampal synaptosomal level was compared when with B6 (*disc1*^+/+^; Figure 2I). Together, these outcomes suggest that pIGF-1Rβ activity, and not the expression, is altered in partial loss of disc1 function (*disc1*^+/−^). On the other hand, decreased IGF-1Rβ expression may be the hallmark of *disc1*^−/−^-related loss of pIGF-1Rβ function.

**Figure 3:**
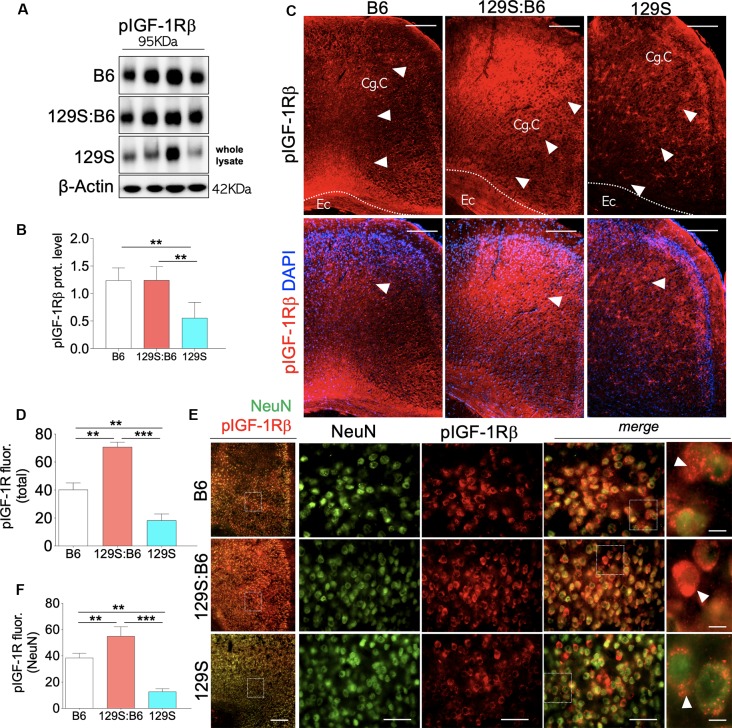
Prefrontal cortex (PFC) expression of pIGF-1Rβ in 129S:B6 and 129S mice. **(A)** Representative western blots for pIGF-1Rβ detection in prefrontal cortical whole tissue lysate. **(B)** One-Way ANOVA comparison of normalized cortical pIGF-1Rβ expression. **(C)** Low magnification immunofluorescence images demonstrating an increase in pIGF-1Rβ expression in the 129S:B6 PFC, and a decrease in the 129S cortex (scale bar = 200 μm; Cg. C: cingulate cortex and Ec: external capsule). **(D)** Bar graph comparing pIGF-1Rβ fluorescence intensity in the PFC. **(E)** Double fluorescence immunolabeling for NeuN/pIGF-1Rβ co-localization in the PFC (scale bar = 200 μm, 60 μm, and 10 μm). **(F)** Quantification of pIGF-1Rβ fluorescence intensity in NeuN-labeled PFC neurons [(*n* = 4 to *n* = 6 per group; **B,D,F**); **p < 0.01 and ***p < 0.001].

Similar to the hippocampus, loss of DISC1 function (*disc1*^−/−^) in the PFC is associated with a decrease in cortical pIGF-1Rβ expression (Figure 3A, also Supplementary Figure S2.1) when compared with 129S:B6 (*disc1*^+/−^, *p* < 0.01) and B6 (*disc1*^+/+^, *p* < 0.01; Figure 3B). In support of this outcome, normalized pIGF-1Rβ fluorescence (Figure 3C, also Supplementary Figures S2.2,S2.3) decreased significantly in the PFC of 129S mice when compared with 129S:B6 (*p* < 0.001) and B6 (*p* < 0.01; Figure 3D). To determine the expression of pIGF-1Rβ in cortical neurons, pIGF-1Rβ immunofluorescence was combined with the NeuN labeling of pyramidal neurons (Figure 3E). In addition to the reduced cortical pIGF-1Rβ fluorescence, the mean expression of pIGF-1Rβ in neurons (NeuN) decreased significantly in the 129S PFC. When compared with the B6 (*p* < 0.01) and 129S:B6 (*p* < 0.001), neurons in the 129S PFC showed a statistical decrease in the pIGF-1Rβ level (Figure 3F).

### pAkt

The kinase activity of pIGF-1Rβ involves the downstream activation of Akt (PBK) through Thr308 phosphorylation (Figure 1). Given that the complete activation of Akt requires Ser473 phosphorylation, here, we evaluated the expression of pAkt (S473) in C57BL/6J, 129S:B6 and 129S brains. Akt expression was determined by normalizing the basal protein level with β-actin. The threshold of Akt activity was determined by normalizing S473 phosphorylated Akt with Akt protein level.

**Figure 4:**
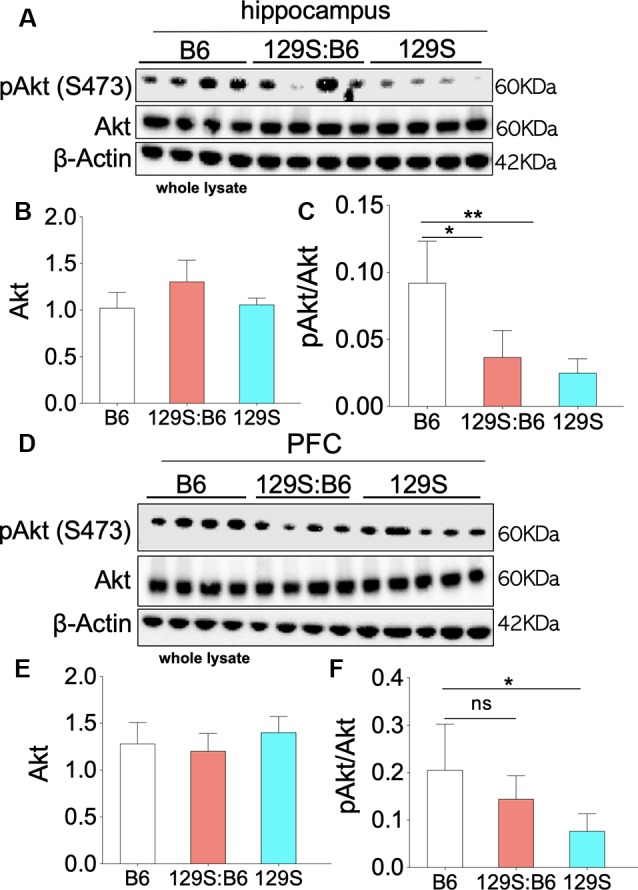
Akt phosphorylation (Ser473) in the 129S:B6 and 129S hippocampus and PFC. **(A)** Western blots demonstrating Akt and *Ser473*-pAkt expression in hippocampal whole lysate. **(B,C)** Bar graphs (One-Way ANOVA) illustrating β-actin normalized Akt expression, and Akt normalized *Ser473*-pAkt expression in hippocampal whole lysate. **(D)** Representative western blots demonstrating normalized Akt and *Ser473*-pAkt expression in prefrontal cortical whole tissue lysate. **(E,F)** Bar graphs representing the relative expressions of Akt and *Ser473*-pAkt in B6 and 129S mutants PFC [(*n* = 4 to *n* = 6 per group; **B,C,E,F**); ns: no significance, *p < 0.05 and **p < 0.01].

Partial (*disc1^+/−^*) and total (*disc1*^−/−^) ablation of DISC1 function did not alter the basal Akt level in the 129S hippocampus (Figure 4A). As a result, no significant change was recorded for Akt expression when 129S was compared with 129S:B6 and B6. Similarly, Akt expression in the 129S:B6 hippocampus did not change significantly vs. the B6 levels (Figure 4B). Although the disc1 ablation did not impact Akt expression, subsequent analysis of Akt activity level revealed otherwise (Figure 4C). The loss of DISC1 function in the 129S hippocampus significantly reduced S473 phosphorylation of Akt when compared with the control (B6; *p* < 0.01). Although the 129S:B6 did not record a decline in pIGF-1R activity or Akt expression, the *disc1*^+/−^ phenotype was also characterized by a reduction of S473 pAkt; compared with B6 level (*p* < 0.05). As such, there was no significant difference in normalized hippocampal S473 pAkt when 129S was compared with 129S:B6 level. Furthermore, both mutant phenotypes (*disc1*^+/−^ and *disc1*^−/−^) recorded a significant loss of S473 pAkt when compared with the B6 (*disc1^+/+^*). Based on these outcomes, loss of S473 pAkt activity in the 129S (*disc1*^−/−^) hippocampus may be directly linked to the decline in hippocampal pIGF-1Rβ activity. However, since the pIGF-1Rβ activity did not reduce in the 129S:B6 hippocampus, loss of S473 pAkt activity might have occurred as a result of other changes directly linked to a defective DISC1 signaling.

Similar to the hippocampus, there was no significant change in basal Akt expression for the *disc1*^−/−^ (129S) and *disc1*^+/−^ (129S:B6) PFC when compared with the B6 group (*disc1*^+/+^; Figures 4D,E). Likewise, the normalized S473 pAkt level decreased significantly in the 129S PFC when compared with the B6 (Figure 4F; *p* < 0.05). An empirical decrease in S473 pAkt was also recorded in the 129S:B6 PFC (Figure 4F). Based on previously established pIGF-1Rβ kinase signaling mechanism (Figure 1), suppression of S473 pAkt in the 129S PFC (Figures 4D–F; *p* < 0.05) agrees with the loss of pIGF-1Rβ activity (Figure 3). Together, these results indicate that pAkt attenuation in the 129S PFC, and not 129S:B6, may be linked to a decrease in pIGF-1Rβ activity.

### GSK3β

GSK3β is involved in several cellular processes that occur downstream of IGF-1Rβ and other tyrosine kinase receptors (Rtks). Unlike IGF-1Rβ and Akt, GSK3β is basally active and does not require phosphorylation to be activated (Dewachter et al., 2009; Hur and Zhou, 2010; Emamian, 2012; Bhat et al., 2018). Mechanistically, phosphorylation of GSK3β (pGSK3β) at the Ser9 site by S473 pAkt attenuates basal GSK3β activity. Here, we used western blotting to detect basal GSK3β expression, and the threshold of Ser9 GSK3β phosphorylation in the hippocampus and PFC. To determine basal GSK3β level, GSK3β was normalized with β-actin. Likewise, the threshold of Ser9 GSK3β phosphorylation was determined by normalizing Ser9 pGSK3β with basal GSK3β.

**Figure 5:**
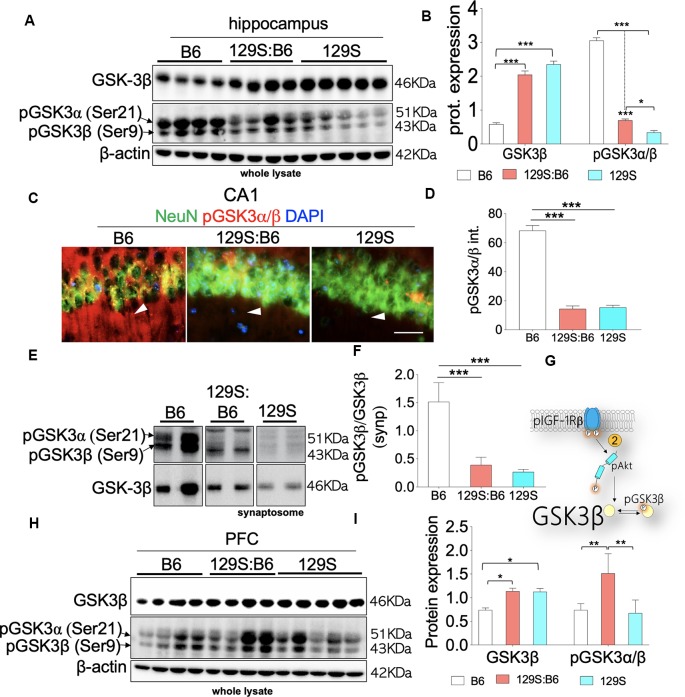
GSK3β activity in the B6, 129S:B6 and 129S brain. **(A)** Representative western blots illustrating the expression of GSK3β and pGSK3β in the hippocampus of B6, 129S:B6 and 129S strains. **(B)** Bar graph illustrating the statistical difference in β-actin normalized GSK3β expression, GSK3β normalized pGSK3β expression in the hippocampus. **(C)** Fluorescence images depicting the loss of pGSK3α(Ser21)/β(Ser9) expression in the 129S:B6 and 129S hippocampal neurons (CA1; scale bar = 60 μm). **(D)** Bar graph illustrating the statistical comparison of pGSK3β fluorescence intensity in the CA1. **(E,F)** Western blots and bar graph illustrating normalized pGSK3β expression in the hippocampal synaptosomal extract. **(G)** Schematic illustration of the pIGF-1Rβ signaling and downstream modulation of GSK3β by Ser473-pAkt. 129S:B6 and 129S hippocampus showed Ser473-pAkt loss and increased basal activity of GSK3β. **(H,I)** Representative western blots and bar graphs illustrating the expression of GSK3β and pGSK3β in the prefrontal cortical whole lysate [(*n* = 4 to *n* = 6 samples per group; **B,D,F,I**); *p < 0.05, **p < 0.01 and ***p < 0.001].

Based on the previously established pIGF-1Rβ kinase signaling mechanism (Dyer et al., 2016), our results revealed that the threshold of Ser9 GSK3β phosphorylation agrees with the S473 pAkt level in the 129S:B6 and 129S hippocampus. Loss of S473 pAkt (Figures 4A–C) in the hippocampus was accompanied by a decrease in Ser9 pGSK3β in the 129S:B6 (*p* < 0.001) and 129S (*p* < 0.001) hippocampus (Figures 5A,B); compared with C57BL/6J. Since GSK3β is basally active, a decrease in the threshold of Ser9 GSK3β phosphorylation indicates an upregulation of GSK3β activity in the 129S:B6 and 129S hippocampus. Accordingly, a decrease in Ser9 GSK3β phosphorylation was accompanied by an upregulation of basal GSK3β level in the 129S:B6 (*p* < 0.001) and 129S (*p* < 0.001) hippocampus when compared with the control (Figures 5A,B). Although there was no significant difference in basal GSK3β level when 129S was compared with 129S:B6 hippocampus, the 129S hippocampus showed a significant decrease in normalized Ser9 pGSK3β vs. 129S:B6 (*p* < 0.05; Figure 5B). The result suggests that the *disc1*^−/−^ phenotype (129S) underlie a higher level of GSK3β activity when compared with *disc1*^+/−^ (129S:B6). The result is further supported by immunofluorescence labeling of pGSK3α/β in the hippocampus (Figure 5C). Fluorescence intensity for immunolabeled pGSK3α/β decreased significantly for the 129S:B6 (*p* < 0.001) and 129S (*p* < 0.001) CA1 when compared with B6 (Figure 5D).

Given that Ser9 pGSK3β is pertinent to synaptic plasticity (Peineau et al., 2007; Clayton et al., 2010; Hur and Zhou, 2010; Lee et al., 2011b), we further compared GSK3β activity in 129S:B6 and 129S hippocampal synaptosomal extracts. Similar to whole lysate immunoblot outcomes, there is a significant loss of Ser9 pGSK3β in the 129S:B6 (*p* < 0.001) and 129S (*p* < 0.001) hippocampal synaptosomal extracts (Figures 5E,F; vs. B6). Based on these results, we determined that basal GSK3β activity is significantly upregulated at the cellular and synaptic levels as a result of *disc1*^+/−^ (129S:B6) and *disc1*^−/−^ (129S) mutation (Figure 5G).

Immunoblot analysis of the prefrontal cortical whole lysate revealed a significant increase in basal GSK3β activity for the 129S:B6 (*p* < 0.05) and 129S (*p* < 0.05) brain (Figures 5H,I). Contrary to the hippocampus, the 129S:B6 cortex showed an increase in normalized Ser9 pGSK3β level (*p* < 0.01; Figure 5I) vs. the C57BL/6J. This indicates a decreased GSK3β activity in the 129S:B6 PFC compared with the control (B6). Interestingly, for the 129S PFC, there was no significant change in Ser9 pGSK3β level vs. the C57BL/6J (Figures 5G–I). From these outcomes, we determine that an increase in prefrontal cortical basal GSK3β activity is a shared attribute of 129S:B6 and 129S PFC. However, the pattern of cortical dysregulation of Ser9 pGSK3β may be dose-specific.

### Erk1/2

**Figure 6:**
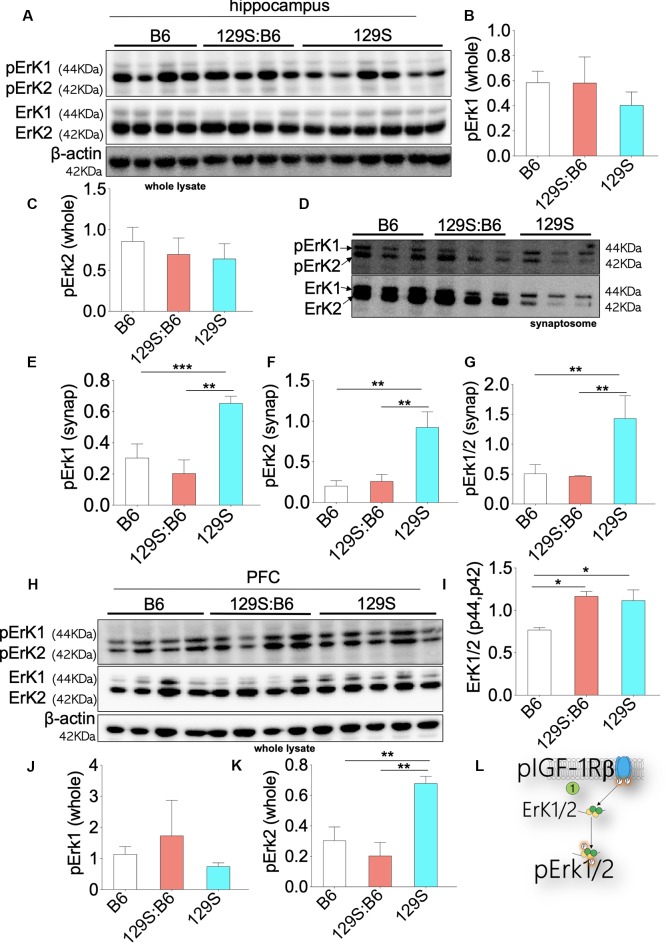
Erk1/2 expression in the B6, 129S:B6 and 129S brain. **(A)** Immunoblots demonstrating the expression of Erk1/2 and pErk1/2 in the hippocampus. **(B,C)** Bar graph illustrating Erk (1 and 2) normalized pErk1 and pErk2 expression in the whole hippocampal lysate. **(D)** Representative western blots illustrating the expression of pErk1 and pErk2 in hippocampal synaptosomal extracts. **(E–G)** Bar graphs representing Erk normalized pErk1, pErk2, and total pErk1/2 expression in hippocampal synaptosomal extracts. **(H)** Immunoblots illustrating prefrontal total cortical Erk1/2 expression. **(I)** Bar graph demonstrating a significant increase in total PFC Erk1/2 expression for the 129S:B6 and 129S mice. **(J,K)** Bar graph representing the expression of Erk normalized pErk1 and pErk2 expression in cortical whole lysate. **(L)** Schematic illustration demonstrating an increase in cortical Erk1/2 expression. [**(E–G,I,K)**; *p < 0.05, **p < 0.01, and ***p < 0.001].

Downstream of pIGF-1Rβ, phosphorylation of Erk1/2 (p42, p44) promotes cell proliferation (Roux and Blenis, 2004; Roskoski, 2012; Xing et al., 2016). This is particularly important in brain development as pIGF-1Rβ and pErk1/2 signaling regulates the distribution of neurons per unit area (Xing et al., 2016; Lin et al., 2017; Nieto Guil et al., 2017). However, given that upstream pIGF-1Rβ/pAkt/GS3β activity is dysregulated in the *disc1*^+/−^ and *disc1*^−/−^ brain, we compared the expression of pErk1/2 in the PFC and hippocampus of these mice strains. The expression of Erk1/2 was normalized with β-actin. To determine Erk1/2 activity (phosphorylation), pErk1 and pErk2 were normalized with the corresponding Erk1 or Erk2 levels. Immunoblot detection of pErk1 and pErk2 in whole hippocampal lysate (Figure 6A) showed that DISC1 loss of function did not alter the overall activity of these proteins. As such, there was no significant difference in pErK1 and pErK2 level when 129S (*disc1*^−/−^) was compared to 129S:B6 level (*disc1*^+/−^). Similarly, the 129S:B6 levels were not significantly different when compared with B6 (*disc1^+/+^*; Figures 6B,C).

In subsequent analysis, we determined the synaptic activity of Erk by detecting pErk1/Erk1 and pErk2/Erk2 in hippocampal synaptosomal extracts (Figure 6D). Although total Erk activity was unchanged in the 129S hippocampus (Figures 6A–C), there was a significant increase in synaptic pErk1/pErk2 activity. When compared with the 129S:B6 (*disc1^+/−^*) and B6 (*disc1^+/+^*), the 129S group recorded an increase in normalized pErk1, pErk2, and pErk1/2 levels (Figures 6E–G; *p* < 0.01). It is important to note that the total synaptic Erk1/2 and pErK1/2 protein levels were downregulated by several folds in the 129S mutants, compared with 129S:B6 and B6 hippocampus (Figure 6D).

Immunoblot analysis of whole PFC lysate showed a significant increase in basal Erk1/2 protein level for the 129S:B6 (*p* < 0.05) and 129S (*p* < 0.05) PFC (Figures 6H,I). Analysis of pErk1 and pErk2 activity in the cortex showed some variations when compared with the hippocampus. Similar to the hippocampus, there was no significant change in total pErk1 activity for the 129S PFC; compared with the B6 and 129S:B6 (Figure 6J). Interestingly, the 129S PFC recorded a significant increase in pErk2 activity when compared with B6 and 129S:B6 (*p* < 0.01; Figure 6K). These outcomes suggest that *disc1*^−/−^ mutation impacts pErK2 function in the PFC and not pErk1.

Our results also showed that the heterozygote disc1 mutation (129S:B6) did not impact pErk1/2 activity in the hippocampus and PFC. As such, there was no significant difference in normalized pErk1 and pErk2 levels when we compared 129S:B6 to B6 hippocampus (Figures 6A,B). This was also the case for the synaptic activity of pErk1 and pErk2 (Figures 6D–G). Similarly, prefrontal cortical expression of pErk1 and pErk2 were not significantly different when the 129S:B6 (*disc1*^+/−^) was compared to B6 (*disc1*^+/+^; Figures 6H,K,L).

## Discussion

*Disc1* mutation is an associative cause of human neuropsychiatric disorders linked with schizophrenia, bipolar depression, and some cases of autism (Brandon et al., 2009; Wexler and Geschwind, 2011; Zheng et al., 2011; Gómez-Sintes et al., 2014; St Clair and Johnstone, 2018). *Disc1* mutation alters the activity of GSK3β, Erk1/2, and Akt in the nervous system (Kim et al., 2009; Soares et al., 2011; Aringhieri et al., 2017; Hirayama-Kurogi et al., 2017; Malavasi et al., 2018). Owing to the role of Erk1/2 and GSK3β in the control of cortical organization, synaptic development, and LTP, *disc1* mutation underlies a broad range of synaptic and neuropsychiatric defects. 129S mice show prominent anatomical brain changes, with observable neuropsychiatric phenotypes in postnatal development (Soares et al., 2011; Sultana et al., 2019). Similarly, disruption of neurotrophic cues involving DISC1, Erk1/2 or GSK3β signaling in the C57BL/6J brain disrupts cell migration and cortical lamination patterns (Koike et al., 2006; Ross et al., 2006; Kvajo et al., 2008; Kim et al., 2009; Lee et al., 2011a,b; Namba et al., 2011; Xing et al., 2016; Nieto Guil et al., 2017).

Erroneous regulation of Erk1/2 signaling promulgates cell proliferation abnormalities in the developing cortical circuit. Experimental hyperactivation of Erk1/2 in the cortex caused a significant increase in neuron count, and morphological defects (Morales-Garcia et al., 2014; Xing et al., 2016). To this effect, pharmacological inhibition of Erk1/2 signaling rescued some of the synaptic and behavioral phenotypes associated with developmental neuropsychiatric defects in mice (Lu and Dwyer, 2005; Soares et al., 2011; Pereira et al., 2014; Tassin et al., 2015; Aringhieri et al., 2017; Hirayama-Kurogi et al., 2017; Pucilowska et al., 2018). Similarly, there is evidence that pharmacological inhibition or genetic ablation of neural GSK3β activity rescued dendritic spine and behavioral abnormalities linked to *disc1* mutation, and other forms of schizophrenia (Lee et al., 2011b; Lipina et al., 2011).

In addition to their role in DISC1 signaling, GSK3β and Erk1/2 are controlled by upstream pIGF-1Rβ activity. An important question yet to be addressed is whether a change in expression of these kinases in the *disc1* mutant brain impacts pIGF-1Rβ activity. Based on previously established mechanisms for pIGF-1Rβ signaling (Dyer et al., 2016), we considered the possible link between IGF-1Rβ, Akt, Erk1/2, GSK3β in the* disc1* mutant brain. In addition to a change in the activity of GSK3β and Erk1/2, neural pIGF-1R expression changed in the *disc1* mutant hippocampus and PFC. Analysis of pIGF-1Rβ-GSK3β signaling showed that the decrease in neural pIGF-1Rβ in the 129S hippocampus was accompanied by the suppression of S473 pAkt. Likewise, the loss of S473 pAkt may be linked to a decrease in Ser9 phosphorylation (inactivation) of pGSK3β (Wan et al., 2007; Kitagishi et al., 2012; Levenga et al., 2017). From these outcomes, we deduced that the dysregulation of DISC1-associated proteins (GSK3β/Erk1/2) impacts pIGF-1Rβ activity in the *disc1* mutant brain (Figure 1).

Our results revealed that an increase in basal GSK3β activity, in the hippocampus of mutant *disc1* carrier mice, is associated with the dysregulation of DISC1-GSK3β interaction, and pAkt-mediated regulation of GSK3β. Thus, in both *disc1*^+/−^ and *disc1*^−/−^ brain, there was a decrease in Ser473 pAkt and a reduction in Ser 9 phosphorylation of GSK3β. This indicates an increase in basal GSK3β activity. A similar increase in GSK3β activity was also observed at the synaptic level. Accordingly, 129S:B6 and 129S synaptosomal extracts recorded a decrease in normalized Ser9 pGSK3β level when compared with the control (B6; Figures 5E,F). Like the hippocampus, the PFC of mutant *disc1* carriers also exhibits a significant increase in basal GSK3β expression when compared with the control (B6; Figure 5I). However, the pattern of GSK3β activity was different when the hippocampus was compared with the cortex. While the 129S:B6 (*disc1*^+/−^) showed an increase in normalized Ser9 pGSK3β, the 129S (*disc1*^−/−^) PFC recorded no significant change vs. the control.

The observed prefrontal cortical GSK3β activity may be related to the dysregulation of pIGF-1Rβ-pAkt in the mutant cortex. As shown previously, the* disc1^+/−^* (129S:B6) brain was characterized by an increase in pIGF-1Rβ expression (Figures 3D–F). When compared with the control (B6), the level of normalized S473 pAkt was also unchanged for the 129S:B6 cortex (Figure 4F). Thus, there is a possibility that an increase in pIGF-1Rβ expression promulgates a higher level of Ser9 pGSK3β in the 129S:B6 cortex, compared with the 129S. Interestingly, the loss of pIGF-1Rβ in the *disc1*^−/−^ (129S) cortex (Figure 3D–F), coupled with a decrease in S473 pAkt (Figure 4F) did not affect the Ser9 pGSK3β threshold. These results suggest that change in Ser9 pGSK3β in the 129S (*disc1*^−/−^) PFC may not be directly linked to pIGF-1Rβ dysregulation.

In addition to a defective pIGF-1Rβ-pAkt-GSK3β, Erk1/2 activity is also dysregulated in the hippocampus and PFC of 129S (*disc1*^−/−^) mutant mice. Specifically, there was an increase in pErk1/2 activity in the hippocampus and pErk2 activity in the PFC of 129S mice. Interestingly, increased ErK activity appeared to phenotype-specific. While the 129S (*disc1*^−/−^) recorded an increased synaptic pErk level, there was no significant change in pErk activity for the 129S:B6 (*disc1*^+/−^) brain when compared with the control (B6; Figure 6). Given that a pIGF-1Rβ decreased in 129S hippocampal whole lysate and synaptosomal extracts, it is likely that pErk increase may be associated with other changes linked to disc1 mutation and post-synaptic DISC1 activity. Similarly, increased pErk2 levels in the 129S PFC demonstrate that a change in Erk activity may not be dependent on pIGF-1Rβ. The limitation of the current study is that loss or gain of function experiments have not been performed for Erk, GSK3β, and Akt. Thus, the results are still preliminary and descriptive.

Taken together, these outcomes suggest that the hippocampus and PFC show distinct patterns of kinase dysregulation in *disc1* mutation. Also, the pattern of dysregulation may vary with the mutation dose. While the 129S:B6 showed mostly GSK3β dysregulation, the 129S brain recorded significant changes in GSK3β and Erk1/2.

## Future Directions

Although Erk1/2 and GSK3β inhibitors are now being explored as therapeutic agents in developmental neuropsychiatric disorders, our results suggest that a broader target involving pIGF-1Rβ may be required for effective control of the pathway. The outcome of this study also revealed a general decrease in neural pIGF-1Rβ as a result of *disc1* mutations (Figures 1, 2). This is in agreement with previous studies that explored IGF-1 therapy to attenuate schizophrenia pathophysiology (Venkatasubramanian et al., 2010; Bou Khalil, 2011; Demirel et al., 2014). On the other hand, an increase in pIGF-1Rβ in the 129S:B6 brain suggests that IGF-1Rβ blockers could be explored as therapeutic targets for some *disc1* carriers. In addition to an increase in GSK3β activity, pErk1/2 is also upregulated in the 129S brain. Thus, a combination of GSK3β and Erk1/2 inhibitors could be further explored.

## Summary

In summary, our results show that neural pIGF-1Rβ expression is altered in 129S:B6 and 129S mice compared with C57BL/6J animals. Furthermore, both strains were characterized by a significant change in GSK3β and Erk1/2 expression patterns in the hippocampus and PFC (Supplementary Table S1). We deduce that some of these changes may be directly related to pIGF-1Rβ expression. Thus, targeting IGF-1Rβ in addition to the kinases (GSK3β and Erk1/2) may reduce the phenotypic burden of some developmental neuropsychiatric disorders.

## Data Availability Statement

The raw data supporting the conclusions of this article will be made available by the authors, without undue reservation, to any qualified researcher.

## Ethics Statement

The animal study was reviewed and approved by Louisiana State University School of Veterinary Medicine IACUC Committee.

## Author Contributions

OO and CL designed the experiments. OO, AS and RS conducted the experiments and analyzed the results. OO and CL prepared the manuscript. OO, CL, RS and AS checked the manuscript.

## Conflict of Interest

The authors declare that the research was conducted in the absence of any commercial or financial relationships that could be construed as a potential conflict of interest.

## Supplementary Material

FIGURE S1 Low magnification merged fluorescence images for Figures 2D,E.

FIGURE S2 S2.1: Immunoblots showing the expression of pIGF-1Rβ in the PFC (as presented in Figure 3A) S2.2: pIGF-1Rβ immunofluorescence in the B6, 129S:B6 and 129S mPFC (see also Figure 3C). S2.3: pIGF-1Rβ and NeuN immunofluorescence in the B6, 129S:B6 and 129S mPFC (also Figure 3E; low magnification image).

TABLE S1 Summary of results.
