# Supplementary figures and images for: Disc1 Carrier Mice Exhibit Alterations in Neural pIGF-1Rβ and Related Kinase Expression
Source: Front Cell Neurosci. 2020 May 5;14:94. doi: 10.3389/fncel.2020.00094 (PMC7214624; doi:10.3389/fncel.2020.00094)

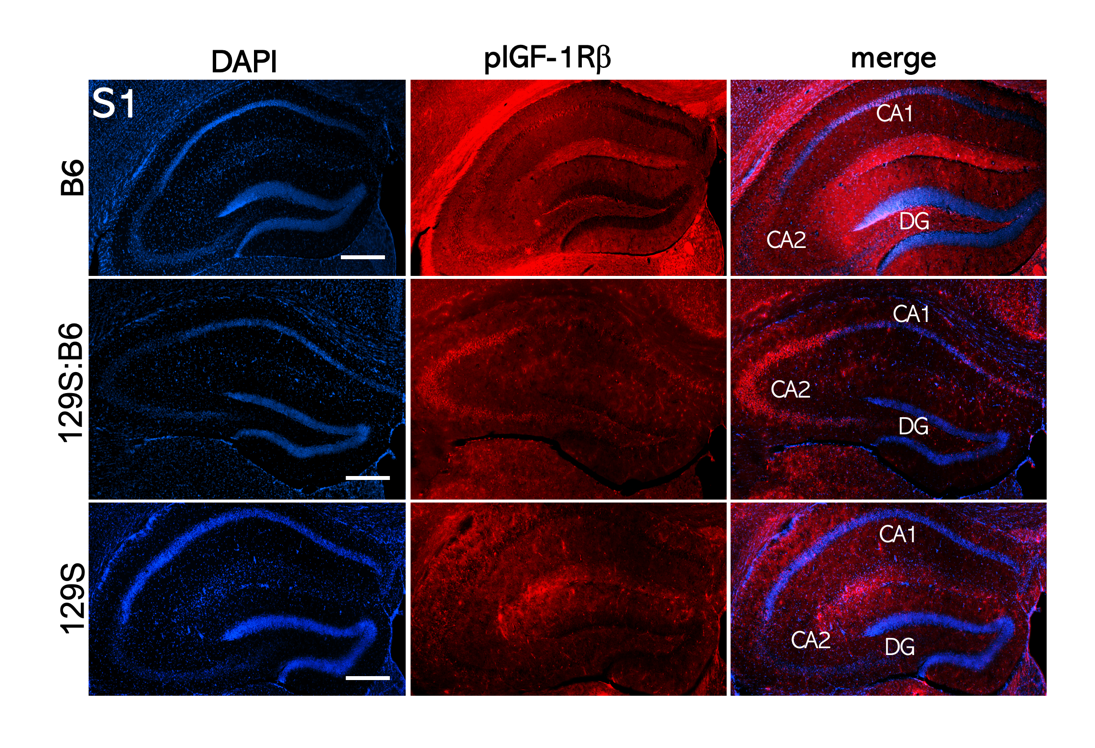

Supplement: FIGURE S1 — Low magnification merged fluorescence images for Figures 2D,E. [file Image_1.TIFF]

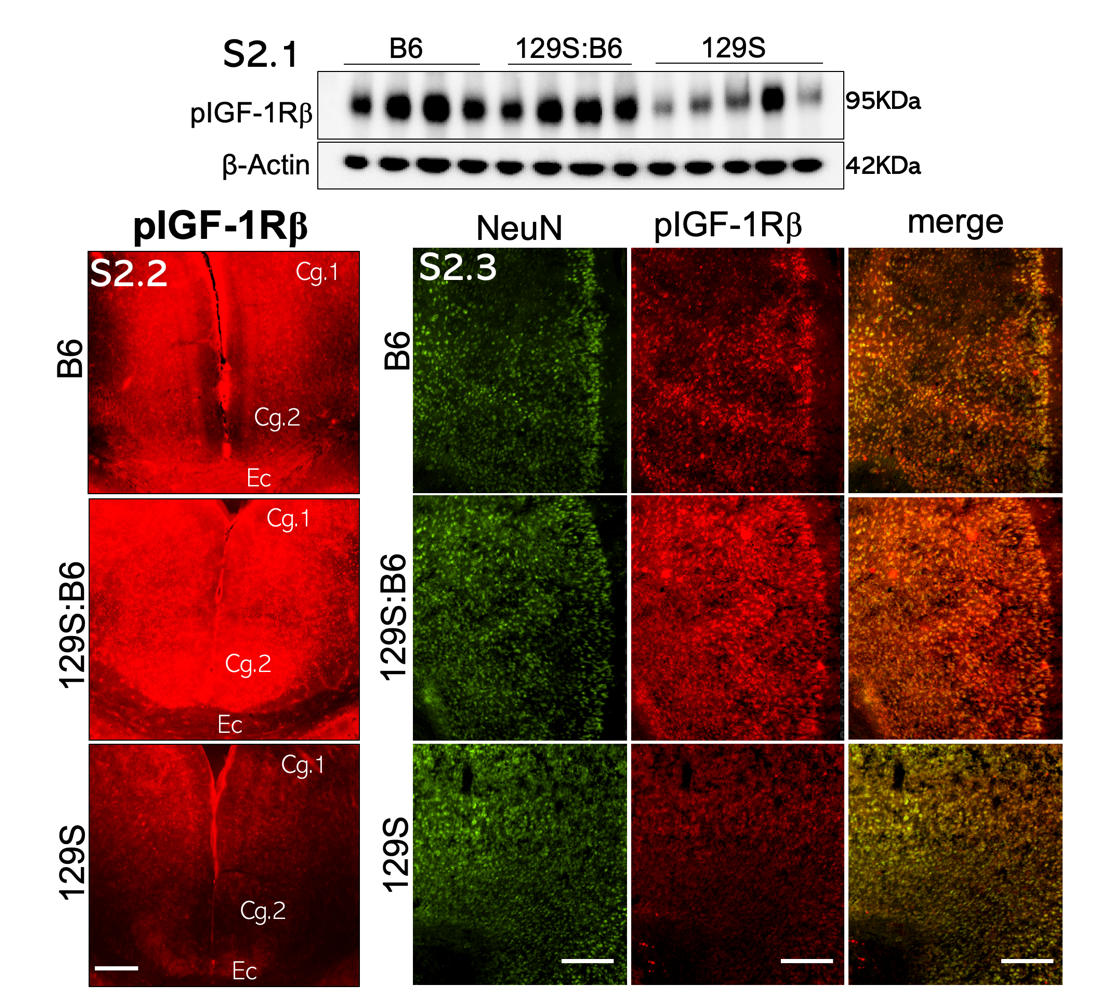

Supplement: FIGURE S2 — S2.1: Immunoblots showing the expression of pIGF-1Rβ in the PFC (as presented in Figure 3A) S2.2: pIGF-1Rβ immunofluorescence in the B6, 129S:B6 and 129S mPFC (see also Figure 3C). S2.3: pIGF-1Rβ and NeuN immunofluorescence in the B6, 129S:B6 and 129S mPFC (also Figure 3E; low magnification image). [file Image_2.TIF]
