# Supplementary material for: Disc1 Carrier Mice Exhibit Alterations in Neural pIGF-1Rβ and Related Kinase Expression
Source: Front Cell Neurosci. 2020 May 5;14:94. doi: 10.3389/fncel.2020.00094 (PMC7214624; doi:10.3389/fncel.2020.00094)
Supplement: TABLE S1 — Summary of results. [file Table_1.docx]

**Table 1:** Summary of results

| **hippocampus** | | **prefrontal cortex** | |
| --- | --- | --- | --- |
| 129S:B6  ***disc1^+/-^*** | 129S  ***disc1^-/-^*** | 129S:B6  ***disc1^+/-^*** | 129S  ***disc1^-/-^*** |
| pIGF-1Rβ↑*** | pIGF-1Rβ↓* | pIGF-1Rβ↑ | pIGF-1Rβ↓ |
| pAkt↓* | pAkt↓** | pAkt (ns) | pAkt↓* |
| GSK3β↑*** | GSK3β↑*** | GSK3β↑* | GSK3β↑* |
| pGSK3β↓*** | pGSK3β↓*** | pGSK3β↑** | pGSK3β (ns) |
| pGSK3β↓*** (synaptosome) | pGSK3β↓*** (synaptosome) |  |  |
| pErk1 (synaptosome) | pErk1↑*** (synaptosome) | pErk1 (ns) | pErk1 (ns) |
| pErk2 (synaptosome) | pErk2↑** (synaptosome) | pErk2 (ns) | pErk2↑** |
| pErk1/2 (synaptosome) | pErk1/2↑** (synaptosome) |  |  |

*↑: increase, ↓: decrease, *p<0.05, **p<0.01, ***p<0.001, ns: no significance*
